# 2513. CERTAIN-1 Subgroup Analysis: A Phase 3 Study of Cefepime-Taniborbactam Efficacy in the Treatment of Complicated Urinary Tract Infections (cUTI)

**DOI:** 10.1093/ofid/ofad500.2131

**Published:** 2023-11-27

**Authors:** Mary Beth Dorr, Leanne Gasink, Tim Henkel, Greg Moeck, Hongzi Chen, Scott A McConnell, Paul McGovern

**Affiliations:** Venatorx, Malvern, Pennsylvania; LBG Consulting, Saint Davids, Pennsylvania; Venatorx Pharmaceuticals, Malvern, Pennsylvania; Venatorx Pharmaceuticals, Malvern, Pennsylvania; Venatorx Pharmaceuticals, Malvern, Pennsylvania; Venatorx Pharmaceuticals, Malvern, Pennsylvania; Venatorx Pharmaceuticals, Malvern, Pennsylvania

## Abstract

**Background:**

Cefepime-taniborbactam (FTB) is an investigational β-lactam/β-lactamase inhibitor combination that is active against carbapenem-resistant Enterobacterales and multidrug resistant *Pseudomonas aeruginosa* expressing serine and metallo-β-lactamases. CERTAIN-1 (Cefepime Rescue with Taniborbactam in cUTI) demonstrated that FTB was superior to meropenem (MEM) for the treatment of cUTI. Subgroup analyses were performed to determine the consistency of response, including for subgroups potentially more challenging to treat (e.g., bacteremia).

**Figure:**
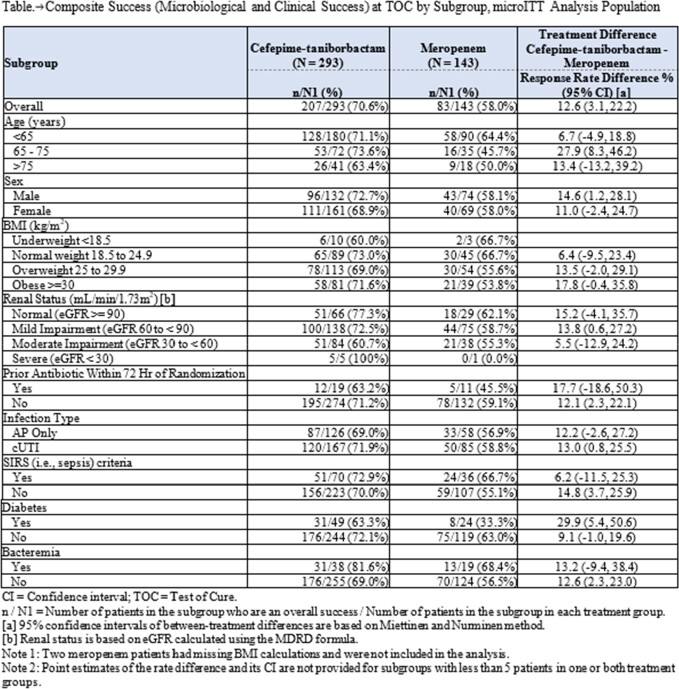

**Methods:**

CERTAIN-1 was a randomized, double blind, double dummy, Phase 3 study comparing FTB to MEM in adults hospitalized with cUTI or Acute Pyelonephritis (AP). The primary endpoint was the composite (microbiologic and clinical) response at the test of cure (TOC) visit in the microITT population. A pre-specified test for superiority for the primary endpoint was performed following confirmation of non-inferiority. Patient demographic and baseline characteristics were analyzed for composite success. Treatment difference in success rate and corresponding 95% CI were calculated.

**Results:**

661 patients were randomized and 436 patients (66.0%) were included in the microITT population; 38.1% of patients were ≥ 65 years of age and 13.1% had baseline bacteremia. Composite success rates were 70.6% and 58.0% for FTB and MEM groups, respectively for the primary endpoint at the TOC visit and FTB was superior to MEM (treatment difference [FTB-MEM], 12.6%; 95% CI, 3.1 to 22.2; p=0.0088). Composite success rates were consistent with the primary analysis for subgroups, including BMI and renal impairment, in patients with potentially more serious infections (e.g., bacteremia, sepsis), and in at-risk patient subgroups (e.g., age ≥ 65, diabetes mellitus) (Table). No subgroup appears to have driven the overall result of superiority of FTB compared to MEM for the composite success rate.

**Conclusion:**

FTB was superior to MEM for the primary endpoint at TOC and composite response rates were numerically higher in all subgroups, consistent with the primary efficacy response.

**Disclosures:**

**Mary Beth Dorr, PhD**, Biomedical Advanced Research and Development Authority (BARDA): Grant/Research Support|Everest Medicines: Grant/Research Support|Global Antibiotic Research and Development Partnership (GARDP Foundation): Grant/Research Support|Merck and Co.: Shareholder|Pfizer: Shareholder|Venatorx Pharmaceuticals, Inc.: Grant/Research Support|Venatorx Pharmaceuticals, Inc.: Employee, stock options and shareholder **Leanne Gasink, MD, MSCE**, Biomedical Advanced Research and Development Authority (BARDA): Grant/Research Support|CSL Behring: Advisor/Consultant|CSL Behring: Consulting fees|Everest Medicines: Grant/Research Support|Evopint Biosciences: Advisor/Consultant|Evopint Biosciences: Consulting fees|Global Antibiotic Research and Development Partnership (GARDP Foundation): Grant/Research Support|LBG Consulting, LLC: Principal|Spero Therapeutics: Advisor/Consultant|Spero Therapeutics: Consulting fees|Venatorx Pharmaceuticals, Inc.: Advisor/Consultant|Venatorx Pharmaceuticals, Inc.: Grant/Research Support|Venatorx Pharmaceuticals, Inc.: Consulting fees|Vera Therapeutics: Advisor/Consultant|Vera Therapeutics: Consulting fees **Tim Henkel, MD, PhD**, Biomedical Advanced Research and Development Authority (BARDA): Grant/Research Support|Everest Medicines: Grant/Research Support|Global Antibiotic Research and Development Partnership (GARDP Foundation): Grant/Research Support|Venatorx Pharmaceuticals, Inc.: Advisor/Consultant|Venatorx Pharmaceuticals, Inc.: Employee, consulting fees, shareholder **Greg Moeck, PhD**, Biomedical Advanced Research and Development Authority (BARDA): Grant/Research Support|Everest Medicines: Grant/Research Support|Global Antibiotic Research and Development Partnership (GARDP Foundation): Grant/Research Support|Venatorx Pharmaceuticals, Inc.: Grant/Research Support|Venatorx Pharmaceuticals, Inc.: Employee, stock options and shareholder **Hongzi Chen, PhD**, Biomedical Advanced Research and Development Authority (BARDA): Grant/Research Support|Everest Medicines: Grant/Research Support|Global Antibiotic Research and Development Partnership (GARDP Foundation): Grant/Research Support|Venatorx Pharmaceuticals, Inc.: Grant/Research Support|Venatorx Pharmaceuticals, Inc.: Employee|Venatorx Pharmaceuticals, Inc.: Stocks/Bonds **Scott A. McConnell, PharmD**, Venatorx: employee|Venatorx: Stocks/Bonds **Paul McGovern, MD**, Biomedical Advanced Research and Development Authority (BARDA): Grant/Research Support|Everest Medicines: Grant/Research Support|Global Antibiotic Research and Development Partnership (GARDP Foundation): Grant/Research Support|Paratek Pharmaceuticals: Shareholder|Venatorx Pharmaceuticals, Inc.: Grant/Research Support|Venatorx Pharmaceuticals, Inc.: Employee, stock options and shareholder

